# Co-Designing a Provincial Newborn Screening Implementation Framework in Pakistan Using a Sequential Blended Workshop Model

**DOI:** 10.3390/ijns12030074

**Published:** 2026-09-11

**Authors:** Aysha H. Khan, Lena Jafri, Dianne Webster, Farkhanda Ghafoor, Tariq Zafar, Afzal Saeed, Jaida Manzoor, Fouzia Ishaq, Tayyaba K. Butt, Saima Zaki, Azeema Jamil, Hafsa Majid

**Affiliations:** 1 Section of Chemical Pathology, Department of Pathology and Laboratory Medicine, The Aga Khan University, P.O. Box 3500, Karachi 74800, Pakistan; aysha.habib@aku.edu (A.H.K.); azeema.jamil@aku.edu (A.J.); hafsa.majid@aku.edu (H.M.); 2 Newborn Metabolic Screening Programme, Lab Plus, Auckland City Hospital, Auckland 1140, New Zealand; dianne.webster@tewhatuora.govt.nz; 3 Roshni Association, P.O. Box 11073, Lahore 54792, Pakistan; fghafoor99@gmail.com; 4 Department of Pediatrics & Neonatology, Fatima Memorial Hospital College of Medicine and Dentistry, Lahore 54000, Pakistan; drtzafar@hotmail.com (T.Z.); tayyabutt@hotmail.com (T.K.B.); 5 Laboratory Services, Sindh Institute of Child Health and Neonatology, Karachi 74800, Pakistan; afzal.saeed@sichn.com.pk; 6 Department of Pediatric Endocrinology and Diabetes, The Children’s Hospital University of Child Health Sciences, Lahore 54600, Pakistan; jaidamanzoor@gmail.com; 7 Department of Pediatrics, Fatima Jinnah Medical University, Sir Ganga Ram Hospital, Lahore 54000, Pakistan; fouziahasnain@hotmail.com; 8 Department of Obstetrics and Gynaecology, Allama Iqbal Medical College, Lahore 54550, Pakistan; saimazaki133@gmail.com

**Keywords:** newborn screening, medical education, education, workshop, screening, low- and middle-income countries, LMICs, policy, implementation framework

## Abstract

Newborn screening (NBS) in Pakistan remains limited by fragmented service delivery, uneven provincial capacity, and the absence of an implementation pathway that links policy intent to operational planning. This study aimed to co-design a provincial NBS implementation framework for Punjab. To do so, we used a sequential blended workshop model supported by visual planning methods. We conducted a qualitative, three-phase co-design process with 19 participants from laboratory medicine, pediatrics, obstetrics, public health, academia, and policy. Phase I consisted of pre-work and an online workshop to establish a shared knowledge base and identify implementation barriers and enablers. Phase II used an in-person, visually facilitated planning workshop to translate these findings into a provincial NBS implementation framework. Phase III involved a asynchronous email-based review to refine the framework and reach consensus. Content analysis from Phase I identified five implementation domains: physical and technical infrastructure, operational systems, clinical integration, workforce and engagement, and governance and policy. In Phase II, these domains were translated into a visual implementation framework that specified priorities, stakeholders, risks, enablers, and action areas for phased rollout. Congenital hypothyroidism was identified as the most feasible entry-point condition. The final framework positioned NBS not as a stand-alone pilot, but as a coordinated provincial system requiring governance, financing, referral pathways, quality assurance, and workforce development. This study contributes an implementation-oriented model for moving from fragmented NBS initiatives to a provincial implementation framework in a low- and middle-income setting. It also offers a practical foundation for broader national scale-up.

## 1. Introduction

Newborn screening (NBS) has evolved into one of the most significant public health interventions of the past half-century because it enables the early detection and treatment of conditions that would otherwise cause severe morbidity and mortality [1]. NBS includes blood spot testing for selected endocrine, metabolic, and other disorders, as well as inspection for visible birth defects and clinical screening for hearing, vision, and congenital heart defects. Its effectiveness depends on more than policy and infrastructure. It also relies on systems for sample collection and transport, laboratory testing, and short- and long-term follow-up, as well as on healthcare professionals who can translate knowledge into practice [2]. Education therefore remains a critical element of NBS system development across the workforce, including pathologists, obstetricians, pediatricians, nurses, midwives, laboratory technologists, and primary care physicians [3,4].

Pakistan’s population exceeds 241.5 million and is growing at 2.55% annually, with projections suggesting it could reach 300 million by 2030 [5]. The total fertility rate is 3.3 births per woman, and approximately 14,700 births occur each day [6]. The economy faces major constraints, including an estimated poverty rate of 22.5% in fiscal year 2025 and projected average inflation of 7.2% for 2026, both of which may affect employment prospects for the country’s large youth population [7,8]. Pakistan’s healthcare system operates through a three-tiered structure across both public and private sectors, with the private sector providing more than 70% of outpatient services. Under the country’s devolved governance arrangement, provincial governments are primarily responsible for healthcare delivery, while the federal Ministry of National Health Services, Regulations and Coordination provides strategic direction and regulatory oversight. Since the 18th Amendment in 2011, provinces such as Punjab and Sindh have managed their own hospitals, budgets, and medical staffing to better address local needs [9]. Public investment in health remains well below the World Health Organization (WHO) recommended threshold, reflecting persistent underinvestment in the sector [10]. Existing social protection and health programs also do not provide a platform for NBS. For example, the Sehat Sahulat Program focuses mainly on inpatient treatment and does not include NBS within its current mandate, while programs such as the Benazir Nashonuma Program prioritize stunting and malnutrition rather than comprehensive biochemical screening at birth [11].

Efforts to establish NBS in Pakistan have developed unevenly and have largely originated from independent initiatives [12,13,14]. The Aga Khan University Hospital (AKUH) located in Karachi in the province of Sindh in Pakistan was the first institution to conduct NBS for congenital hypothyroidism (CH), doing so from 1987 to 1989 [15,16]. Although the card-based program was subsequently discontinued, serum-based screening continued till 2018 with 50–60% coverage. In 2019, it transitioned to a more structured universal NBS program using dried blood spot (DBS) heel-prick testing. This program later expanded to include congenital adrenal hyperplasia and 12 selected inherited metabolic disorders (IMDs) [17]. International partnerships, including support from the International Atomic Energy Agency (IAEA), have contributed to capacity building [18]. However, many regional initiatives remain fragmented and dependent on external funding. Some provincial progress has also occurred, including the Sindh Newborn Screening Act and non-government organization-led screening initiatives, but a cohesive national NBS infrastructure is still lacking. The Asia-Pacific Society of Human Genetics (APSHG) Working Group on NBS has continued to support countries in the region that lack national programs and have less than 50% screening coverage, including Pakistan. Through workshops and information-sharing activities, the group has helped participants navigate implementation barriers and explore more sustainable approaches to system development [19].

In Punjab, several CH pilot projects have been launched, but none has achieved district-level implementation, and limited services continue only in selected hospitals in Lahore [20,21,22]. In Khyber Pakhtunkhwa and parts of Punjab, a non-government organization-led initiative that began in 2016 screened more than 69,000 newborns and identified 59 CH cases [15]. In Sindh, policy commitment was established through the Sindh Newborn Screening Act of 2013. This was followed by a CH pilot at the National Institute of Child Health from 2019–2022 and its later transition to the Sindh Institute of Child Health and Neonatology for province-wide expansion [23]. Most of these projects relied heavily on international funding for screening equipment and reagents. As a result, many remained limited to research settings or individual hospital practices rather than becoming integrated public health services. The main constraints were the lack of dedicated government infrastructure, insufficient human resources, the absence of a national policy, and the lack of a sustainable provincial financing framework.

Against this background, the present study was designed to address a practical gap in Pakistan’s NBS landscape: the absence of a structured implementation framework that can translate dispersed expertise, advocacy, and pilot experience into a coordinated provincial plan. Using a sequential co-design process delivered through a blended workshop model and informed by visual planning methods, we worked with multidisciplinary stakeholders to develop a provincial NBS implementation framework for Punjab. The study’s contribution is therefore not the description of barriers alone, but the creation of an implementation-oriented framework that organizes those barriers into actionable domains, priorities, and next steps for system development.

## 2. Materials and Methods

### 2.1. The Study Design

This study used a sequential qualitative co-design process delivered through a blended workshop model. The process combined pre-session reading, an online workshop, an in-person planning workshop, and asynchronous consensus-based refinement. It was intended to move participants from a shared understanding of NBS concepts and system barriers to collaborative development of a provincial NBS implementation framework for Punjab.

### 2.2. Study Site

The two hours online workshop was held using the Aga Khan University’s licensed Zoom platform (Zoom Video communication, Inc., USA) and the half-day, in-person workshop was held at Jinnah Hospital, Lahore, Pakistan, in collaboration with Aga Khan University, Karachi, Pakistan.

### 2.3. Study Participants and Data Collection

The study was conducted in three sequential phases with a consistent participant cohort. Nineteen individuals took part, including participants and facilitators from laboratory medicine, pediatrics, obstetrics, public health, academic medicine, and policy-related roles across multiple institutions. The same core group contributed across phases to support the continuity of discussion and iterative refinement. The blended workshop model combined flipped pre-learning materials with interactive group work and was designed to generate both qualitative data on implementation needs and a consensus-based implementation framework.

### 2.4. Phase I Pre-Work and Online Workshop

In Phase I, participants received curated pre-reading materials ten days before the workshop, including international guidance on neonatal dried blood spot screening and literature on NBS implementation in low- and middle-income countries (LMICs). The online workshop, conducted via Zoom in August 2025, established a shared conceptual foundation and elicited participants’ views on the policy context, implementation barriers, and local service realities in Punjab. Facilitated discussions and small-group activities focused on what would be required to establish a provincial NBS system and which constraints were most likely to affect implementation.

### 2.5. Phase II Development of the Implementation Framework

Phase II consisted of a half-day in-person workshop held in Lahore in September 2025. This phase shifted from problem identification to framework development. Using a study-specific visual planning template informed by Grove visual planning methods [24], participants worked individually and in groups to organize implementation needs into a provincial NBS implementation framework. Activities focused on stakeholder mapping, prioritization of tasks, identification of risks and enablers, articulation of short- and medium-term goals, and definition of the operational conditions required for NBS rollout. The workshop output was a consensus-derived visual framework that translated qualitative findings from Phase I into an implementation-oriented planning tool.

### 2.6. Phase III Asynchronous Consensus on the Implementation Framework

In Phase III, the draft framework generated during the in-person workshop was circulated to all contributors for asynchronous review by email. Participants were invited to comment on accuracy, completeness, prioritization, and feasibility. Feedback from this round was incorporated to refine the framework and consolidate it into a stakeholder-informed provincial NBS implementation framework without requiring additional synchronous meetings.

### 2.7. Data Analysis

Facilitators documented discussions, workshop notes, and participant-generated planning outputs across the three phases. Data from Phase I were analyzed using pragmatic content analysis to identify recurrent implementation themes relevant to the study objective. Similar concepts were grouped into categories, which were then reviewed against participant inputs during later phases. These categories informed the structure of the visual planning exercise in Phase II and the refinement process in Phase III.

## 3. Results

Participants represented a multidisciplinary group that included laboratory professionals, pediatricians, obstetricians, policymakers, academic leaders, and allied health contributors. This mix of roles allowed the workshops to address NBS as both a technical service and a system-level public health intervention.

Phase I generated the descriptive foundation for the implementation framework. Through facilitated discussion, case-based reflection, and small-group activities, participants identified the main system components required for a provincial NBS program and the principal barriers to establishing them in Punjab. Content analysis yielded five interrelated implementation domains: physical and technical infrastructure, operational systems, clinical integration, workforce and engagement, and governance and policy. These domains provided the analytic structure for subsequent planning and are summarized in Table 1.

**Figure 1:**
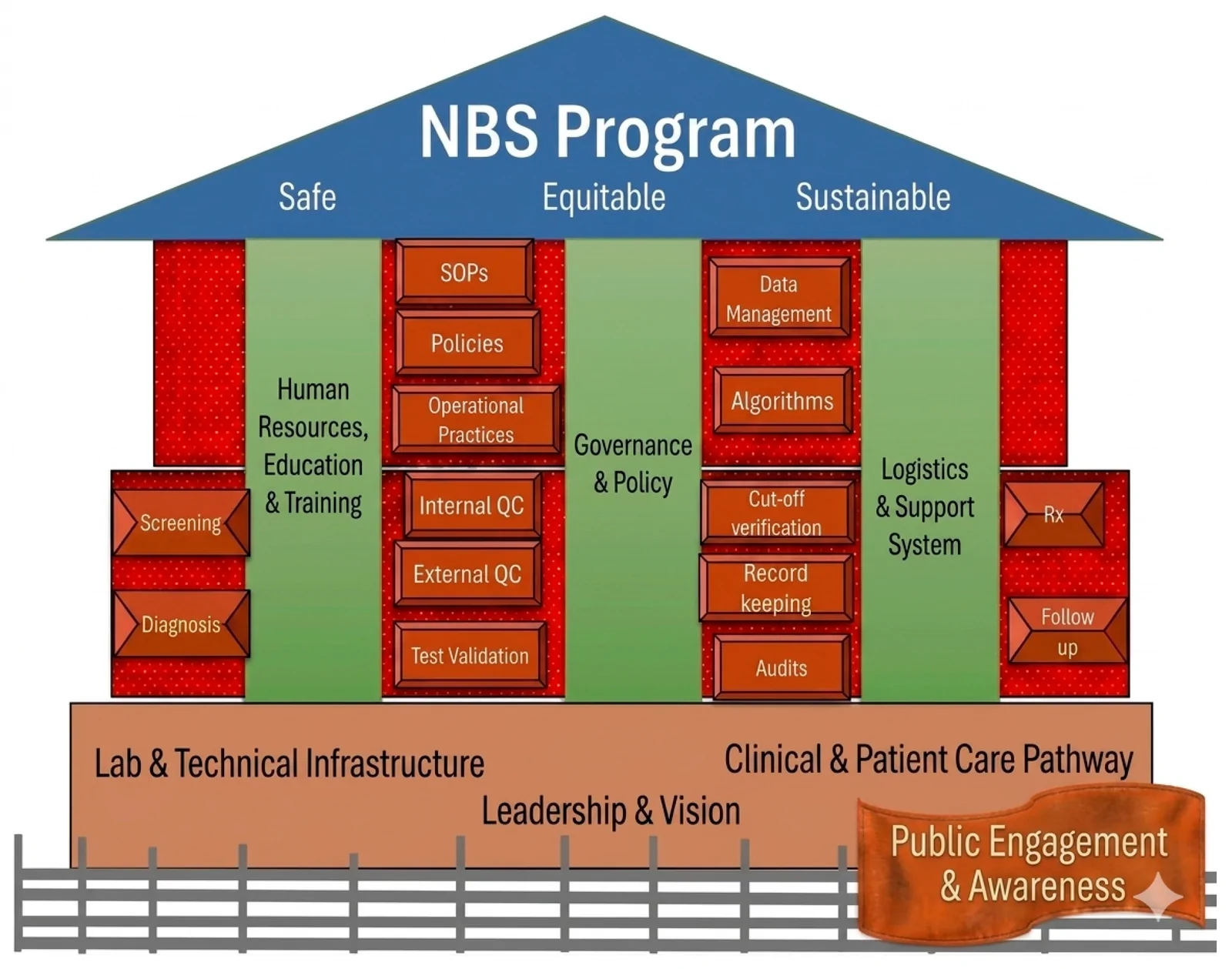
The ‘NBS House’ depicting foundations, pillars, and supporting bricks for implementing NBS in Punjab co-designed by workshop participants.

A second output of Phase I was the co-development of an “NBS house” model (Figure 1), which participants used to represent the internal structure of a provincial NBS system. In this model, foundational readiness underpinned the system, key operational and regulatory functions were represented as pillars, and specific implementation components were expressed as supporting “bricks.” Cross-cutting system safeguards, including patient safety, were positioned at the roof level, while family education and engagement were conceptualized as the external boundary surrounding the model. The diagram served as a shared visual synthesis of participant thinking and informed the more detailed planning work undertaken in Phase II.

Phase II translated these descriptive findings into an implementation-oriented framework. Using the visual planning template, participants identified priority tasks, anticipated barriers, enabling conditions, institutional assets, and expected outcomes for provincial rollout. The resulting framework linked specific interventions to desired system changes and clarified how implementation would depend on coordination across policy, laboratory services, clinical pathways, logistics, training, and public engagement. Across groups, participants consistently identified CH as the most feasible entry-point condition for an initial provincial rollout because of its clinical importance, established treatment pathway, and relative suitability for phased implementation. Participants also emphasized that early success would depend not only on laboratory capacity but on clear referral pathways, multidisciplinary leadership, workforce training, and sustained policy support. Notes, flip charts, and framework drafts from the workshop were carried forward into Phase III for consolidation.

**Figure 2:**
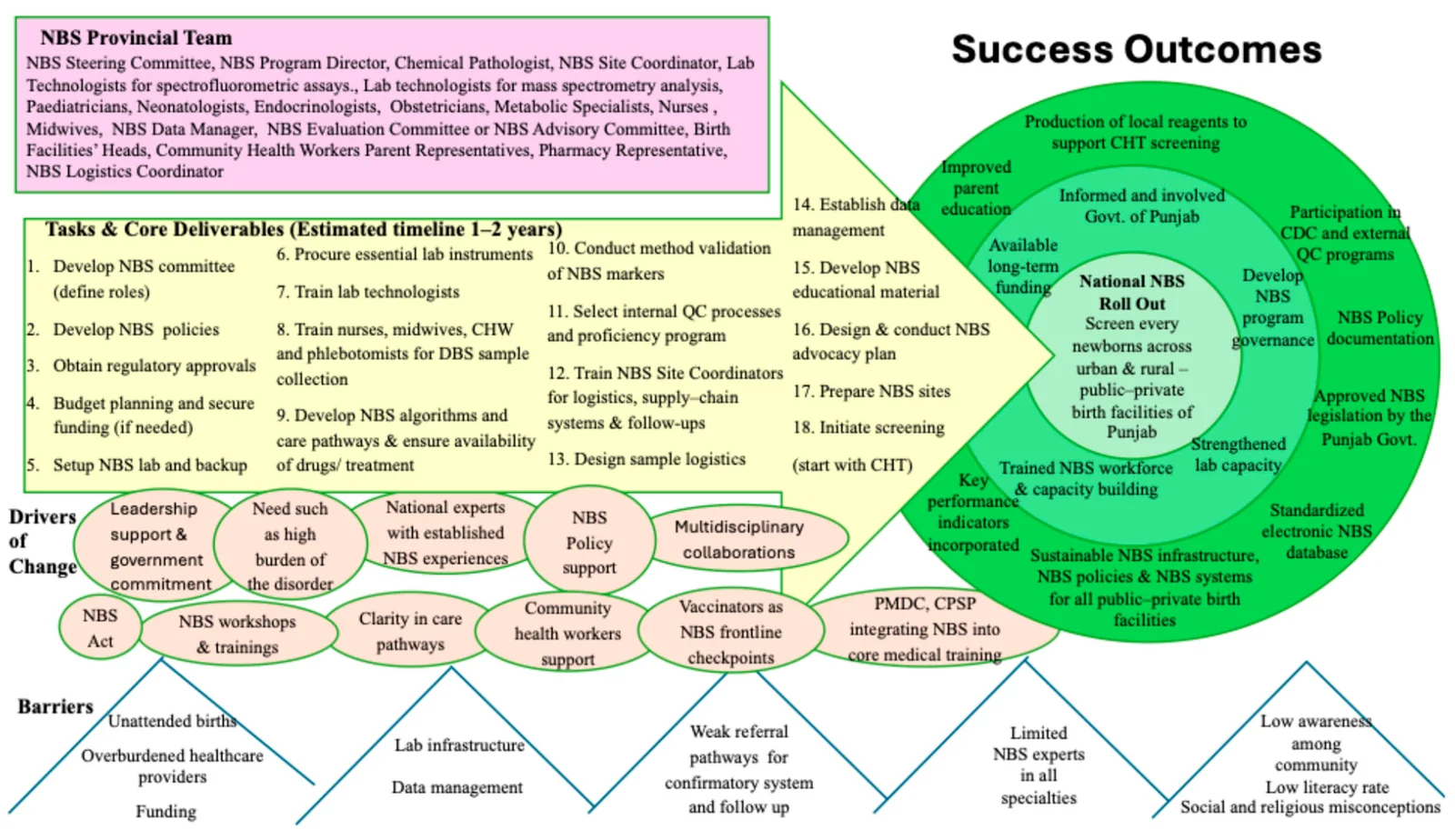
Provincial newborn screening implementation framework for Pakistan.

Inputs from both workshops (Phases I and II) and subsequent email discussions (Phase III) were iteratively reviewed and consolidated into an actionable framework as summarized in Figure 2.

Following asynchronous review and consensus refinement, inputs from all three phases were consolidated into the final provincial implementation framework (Figure 2). The process also resulted in the identification of contributors willing to support further implementation planning, leading to the formation of an initial NBS core group.

## 4. Discussion

Globally, newborn bloodspot screening programs vary considerably in their availability, coverage, scope, and infrastructure, with substantial implementation gaps persisting across countries, particularly in settings where national programmes remain limited or fragmented [25]. Against this broader global context, the study findings from Pakistan show how a sequential co-design process can convert dispersed stakeholder experience into a structured planning tool. This study contributes an implementation framework for provincial NBS in Pakistan. The value of the approach lies less in the workshops themselves than in what they produced: a shared implementation logic linking barriers, priorities, stakeholders, and action areas. In a setting where NBS efforts have often remained localized, donor-dependent, or institution-specific, the framework offers a way to move from fragmented initiatives toward coordinated provincial planning.

The findings also suggest that the principal challenge in Punjab is not the absence of interest in NBS. Rather, it is the absence of an organizing structure capable of aligning governance, technical capacity, referral systems, and financing. This is why the framework matters at the policy level. Its domains correspond closely to the system functions that must be coordinated if NBS is to progress beyond isolated pilots. In this sense, the study aligns with the 77th World Health Assembly resolution (WHA77.17), which calls for accelerated action on NBS and birth defect management in LMICs. It also shows why such action must be adapted to provincial realities rather than imported as a fixed model [26].

The identification of CH as the preferred entry-point condition is analytically important. It shows how participants prioritized feasibility alongside clinical value. A phased start with CH offers a realistic pathway for early implementation in Punjab. However, the workshops made equally clear that screening cannot succeed as a laboratory activity alone. The most persistent barriers identified across phases were shortages of trained personnel, weak follow-up arrangements, fragmented care pathways, and the lack of clear accountability across institutions. Similar implementation constraints have been reported in other LMIC settings, including Africa, Bangladesh, and Indonesia [27,28,29,30]. The framework therefore supports a phased strategy. In this strategy, disorder selection, workforce preparation, transport systems, reporting pathways, and specialist follow-up are developed as interdependent components rather than as separate workstreams.

The framework is also relevant to broader maternal and newborn health priorities in Pakistan, including Every Newborn Action Plan, because it positions NBS within existing public health structures rather than outside them [31,32]. In Punjab, the Integrated Reproductive, Maternal, Newborn and Child Health and Nutrition Programme, Government of Punjab, Pakistan provides an established platform for delivery of maternal and newborn health services through government health facilities and community-based services. Advocacy for inclusion of NBS within such programs could therefore provide a pragmatic pathway for financing and scaling NBS. Rather than seeking funding for NBS as an isolated laboratory service, the program could be presented as an essential preventive health intervention, with the cost incorporated into existing maternal and newborn healthcare packages. This approach could facilitate equitable access, while simultaneously creating a mechanism for government ownership and long-term sustainability.

Integration into existing platforms such as community health worker networks, lady health workers (LHW) or immunization services is only plausible if additional responsibilities are matched with resources, training, and referral capacity [33,34]. Without this, integration risks producing symbolic coverage rather than effective follow-up, particularly in rural and underserved areas The practical implication is that provincial scale-up will require deliberate sequencing, investment, and governance arrangements rather than simple attachment to already burdened programs.

Long-term sustainability in NBS requires government ownership and sustained public financing. Experience in Pakistan shows that externally funded initiatives often end when donor support ceases, highlighting the need to embed NBS within provincial government health systems. The study findings emphasize that financing should be transparent, accountable, and goal oriented, with international support complementing and not replacing government responsibility for equitable access. Pakistan’s previous IAEA-supported CH screening initiative offers a useful foundation. It strengthened training, technical expertise, blood spot TSH screening, and locally adapted assay capacity [16]. This experience underscores the importance of converting successful externally supported pilots into government-owned programs backed by domestic financing. Rather than building new systems from the ground up, Pakistan could revisit, strengthen, and scale this existing experience, particularly locally developed and potentially lower-cost screening approaches, within a government-led national NBS strategy. A further implication of this study is that implementation progress in Pakistan will depend on stronger bridges between advocacy, professional education, and operational planning. Over the past decade, academic institutions, professional societies, and rare disease networks have expanded awareness of NBS and inherited metabolic disorders in Pakistan [35,36,37,38,39]. What has been less developed is a mechanism for converting this momentum into a coordinated service model. The framework presented here helps fill that gap. It offers a planning structure that professional bodies and provincial stakeholders can use to organize roles, define standards, and support implementation beyond individual projects.

This study has several limitations. First, the visual framework is intended as a strategic planning tool. It does not replace the technical guidance required for assay validation, laboratory quality systems, financing models, or long-term follow-up. Second, the participant group was small and purposive. It was also drawn from a limited number of institutions within a single provincial context, which constrains transferability. Third, because some members of the research team had prior experience in NBS implementation, interpretive bias is possible. However, efforts were made to maintain transparency and incorporate participant review during framework refinement. Another limitation was that families and parent representatives were not directly engaged as stakeholders in this project. This may have limited our understanding of family level barriers to NBS awareness, access and follow-up. Future work should incorporate family perspectives to inform culturally appropriate outreach, education and short- and long-term follow-up strategies. These limitations should be considered when interpreting the framework, which is intended to guide implementation rather than provide a detailed operational plan.

### Roadmap for Action

The framework developed in this study implies a phased implementation pathway rather than immediate province-wide rollout. The following actions are the most important next steps for translating the framework into practice.

- A multidisciplinary NBS core group with a defined provincial mandate can provide strategic leadership and coordination. This group may consist of representatives from neonatology, chemical pathology, public health administration, nursing, and health policy, and should be tasked with setting priorities, coordinating institutions, defining governance arrangements, and serving as the main liaison with provincial health authorities. Over time, this provincial structure could provide the basis for a broader national taskforce.
- A key priority for implementation should be the development of a sustainable financing mechanism led by the government, with NBS incorporated into national and provincial maternal, neonatal and child health priorities and supported through recurrent public sector funding. Where appropriate, existing government-funded health programs and service packages could provide platforms through which NBS can be incorporated, rather than establishing parallel financing and service delivery structures.
- Promote an accountable NBS program, with clearly defined responsibilities and objectives, transparent procurement and budgeting, independent monitoring, regular financial and program audits, and measurable performance indicators to ensure that resources reach the intended population and goals are achieved. Implementation can begin with a time-limited pilot project focused on CH in two contrasting districts, such as one urban and one rural setting. Early funding can be used to establish laboratory infrastructure, demonstrate feasibility, generate local epidemiological and health–economic evidence, and develop the workforce and referral pathways required for a national program. The pilot project can evaluate logistics, sample transport, laboratory workflow, reporting, referral, and follow-up feasibility. A phased pilot would generate operational evidence for wider provincial scale-up.
- Mapping existing provincial capacity across laboratory infrastructure, referral pathways, workforce readiness, transport systems, and opportunities for integration with current maternal and child health services can identify gaps and strengths. This can be followed by a resource and partnership strategy identifying start-up needs, in-kind contributions, grant opportunities, and institutional collaborations.
- Endorsement and technical support from relevant professional bodies and training institutions can strengthen scientific credibility, support standard setting, align multidisciplinary training, and improve the likelihood of policy uptake. Continued active participation in the Asia Pacific Society of Human Genetics Working Group on Consolidating Newborn Screening Efforts in the Asia Pacific Region should be prioritized to facilitate knowledge exchange and shared learning with neighboring countries facing similar challenges in developing national NBS programs. Engagement with existing maternal and child health platforms can be pursued selectively and only where referral capacity, supervision, and financing can support meaningful implementation.
- At the national level, we propose the establishment of a Pakistan NBS Ambassador Network, with representatives from each province and relevant national stakeholders, to build a coordinated national advocacy platform for NBS. This will facilitate resource mobilization, promote evidence-based policy development, and strengthen engagement with international partners. Ambassadors from each province could identify existing government-funded maternal and child health programs within their respective provinces and advocate for the inclusion of NBS as a funded component of those programs.

## 5. Conclusions

This study presents a co-designed provincial NBS implementation framework for Punjab, Pakistan, and argues that the central challenge is not whether NBS is valuable, but how it can be organized as a coordinated public health system. By converting stakeholder knowledge into a structured implementation framework, the study provides a practical bridge between fragmented initiatives and system-level implementation. If supported by governance, financing, workforce development, referral pathways, and phased piloting, this framework can serve as a realistic foundation for provincial scale-up and, ultimately, wider national adoption.

## Figures and Tables

**Table 1:** Content analysis from discourse on NBS implementation in Punjab Pakistan during the online workshop in first phase of the study.

Category	Key Elements and Requirements
Physical & Technical Infrastructure	Lab & Equipment: Dedicated NBS Lab (BSL 2); pre-analytical supplies for screening and confirmatory tests; analytical platforms (FIA, ELISA, GCMS, LCMSMS, HPLC) with backup systems. Data & IT: Secure servers, LIS/HIS connectivity, audit trails, and barcode systems for tracking. Post-Analytical: Secure result transmission, integrated reporting with birth facilities, and dedicated recall/communication channels for families and providers.
Operational Systems	Logistics & Transport: Systems for DBS collection (vacutainers, drying racks); secure, temperature-controlled transport (cold chain for serum/plasma); chain-of-custody documentation via courier or dedicated vehicles. Management & Quality: Inventory forecasting, reagent buffer stocks, and equipment maintenance plans. Quality Management System (QMS): Internal/external quality controls, proficiency testing, method validation, and KPI audits.
Clinical Integration	Preparedness: Training for obstetric, neonatal, and nursing staff; maternity workflow integration; and parent education/consent processes. Clinical Pathways: Established screening algorithms and verified cut-offs; interpretation by chemical pathologists; and structured notification for positive results. Care Management: Defined referral pathways to endocrinologists/metabolic physicians, pharmacy coordination for therapies, and genetic counseling.
NBS Workforce and Engagement	Staffing: NBS leadership, coordinators, site supervisors, and lab personnel trained in troubleshooting and all testing phases. Training: National online modules, SOP-based competency training, and outreach via MCH. services. Community: Public awareness campaigns (mass media, brochures); engagement with religious/community leaders and parent support groups; and outreach to underserved areas.
NBS Governance and Policy	Oversight: Provincial/Regional Steering Committees and Technical Advisory Groups with defined accountability. Policy Framework: National-provincial guidelines for mandatory vs. voluntary screening; ethical guidelines for privacy; and alignment with ISNS/WHO standards. Sustainability: Dedicated government funding for start-up and operations; inter-departmental Memorandum of Understanding; collaboration with professional societies; and public health reporting via national dashboards.

Adapted from the first phase of a content analysis study regarding newborn screening implementation in Punjab, Pakistan. Abbreviations: BSL 2, Biosafety Level 2; DBS, Dried Blood Spot; FIA, Fluorescence Immunoassay; ELISA, Enzyme-Linked Immunosorbent Assay; GCMS, Gas Chromatography–Mass Spectrometry; LCMSMS, Liquid Chromatography Tandem Mass Spectrometry; HPLC, High-Performance Liquid Chromatography; LIS/HIS, Laboratory/Hospital Information Systems; SOP, Standard Operating Procedure; QMS, Quality Management System; KPI, Key Performance Indicators; ISNS, International Society for Neonatal Screening; WHO, World Health Organization; MCH, Maternal and Child Health.

## Data Availability

The qualitative data supporting the findings of this study are fully integrated within the text of the manuscript. No additional raw datasets were generated or analyzed during this study.

## Abbreviations

The following abbreviations are used in this manuscript:

NBS	Newborn Screening
WHO	World Health Organization
AKUH	Aga Khan University Hospital
CH	Congenital Hypothyroidism
DBS	Dried Blood Spot
IMD	Inherited Metabolic Disorders
IAEA	International Atomic Energy Agency
APSHG	Asia-Pacific Society of Human Genetics
Pak-IMD-Net	Pakistan Inherited Metabolic Disorders Network
SNARE	Society for Novel and Rare Diseases
WHA77	77th World Health Assembly resolution (WHA77.17)
LMIC	Low- and middle-income countries
LHW	Lady Health Workers
ENAP	Every Newborn Action Plan
IFCC	International Federation of Clinical Chemistry and Laboratory Medicine
ISNS	International Society for Neonatal Screening
PSCP	Pakistan Society of Chemical Pathology
PPA	Pakistan Pediatric Association
